# Adolescent Empathy Influences Bystander Defending in School Bullying: A Three-Level Meta-Analysis

**DOI:** 10.3389/fpsyg.2021.690898

**Published:** 2021-08-05

**Authors:** Xiaoping Deng, Junru Yang, Yuzhen Wu

**Affiliations:** ^1^Research Center of Educational Economics, Gannan Normal University, Ganzhou, China; ^2^School of Education Science, Gannan Normal University, Ganzhou, China; ^3^Department of Rear-Service Management, Gannan Normal University, Ganzhou, China

**Keywords:** bullying, empathy, bystander defending, three-level meta-analysis, cognitive empathy, affective empathy, adolescents

## Abstract

Even though numerous studies have shown that adolescent empathy is positively related to bystander defending in school bullying, others have failed to detect a significant association between these two variables. To address this discrepancy, a three-level meta-analysis of 27 papers (35 independent studies, *N* = 25,012 adolescents) was conducted. The results showed that empathy was positively correlated with bystander defending. Furthermore, the strength of the relationship between empathy and bystander defending was moderated by the type of empathy and the evaluators of defending. Specifically, the correlation coefficient between affective empathy and bystander defending (*r* = 0.27, 95% CI [0.22, 0.32]) was significantly stronger than that between cognitive empathy and bystander defending (*r* = 0.22, 95% CI [0.17, 0.28]). Finally, the strength of the relationship between empathy and bystander defending was moderated by the evaluator of defending behavior. That is, the correlation coefficient of bystander defending measured by self-evaluation was significantly stronger than that measured by peer-evaluation. The results showed that empathy was closely related to bystander defending. Thus, school bullying can be prevented from the perspective of enhancing empathy among adolescents.

## Introduction

School bullying is a predominant social issue worldwide and refers to repeated attacks that cause physical and psychological harm to victims (Olweus, 2013). Research by Zhang et al. (2018) showed that 27% of junior high school students in China have experienced school bullying. School bullying has serious adverse effects for all involved. For example, victims have lower academic achievement and are likelier to experience loneliness, depression, anxiety, and suicidal ideation (Nakamoto and Schwartz, 2011). Bystanders feel guilt and shame, causing loss of confidence and lower self-esteem (Mazzone et al., 2016). Furthermore, bullying behavior increases the risk of bullies perpetrating crimes in adulthood (Klomek et al., 2015).

Previous empirical studies show that the behavioral reactions of bystanders greatly influence school bullying. Approximately 74 percent of the individuals involved in bullying are bystanders (Pouwels et al., 2017). Bystander defending behavior can effectively end bullying behavior (Hawkins et al., 2001) and reduce the incidence of school bullying. Bystander defending refers to the behaviors that are carried out to support the victim, such as comforting the victim in the bullying situation, seeking help from adults or others, resisting the bully, and so forth (Salmivalli et al., 1996). Therefore, promoting bystander defending is essential for the intervention and prevention of school bullying, and researchers are increasingly focusing on interventions for school bullying via bystander defending. However, only 19% of bystanders engage in defending behavior (Hawkins et al., 2001).

### Empathy and Defending

Furthermore, previous research indicates that students with a high level of empathy are likelier to engage in bystander defending during school bullying situations (Cuervo et al., 2018). Most studies define empathy as understanding other people's emotions and sharing their emotional states (Davis, 1983; Jolliffe and Farrington, 2004). Currently, empathy is predominantly measured using the Interpersonal Relation Index (IRI) compiled by Davis (1983). It comprises 28 items, including four dimensions: perspective taking, empathic concern, fantasy, and personal distress. Baron-Cohen and Wheelwright (2004) developed the Empathy Quotient (EQ) for abnormal groups (such as people with autism). It comprises 60 items including cognitive empathy, emotional reactivity, and social skills. Jolliffe and Farrington (2006) suggested that the previous scales failed to distinguish sympathy from empathy and to accurately measure cognitive empathy. To address these limitations, they compiled the Basic Empathy Scale (BES) which consists of 20 items, including two dimensions of cognitive empathy and affective empathy.

However, previous studies regarding the relationship between empathy and bystander defending yielded conflicting results. For example, both the empathy-altruism hypothesis (Batson, 1987) and the theory of prosocial moral behavior and development (Hoffman, 2001) propose that witnessing another person in distress stimulates an empathic response, guilt, anger, and a desire to alleviate the distress, which results in helping behavior. Bystander defending is altruistic in a specific situation; that is, when bystanders witness school bullying, they will empathize with the painful experience of the victim, thereby prompting bystanders to engage in defending behavior. Several empirical studies have supported this view and found that empathy has a significant positive correlation with bystander defending (Gini et al., 2007, 2008; Nickerson et al., 2008; Xie and Ngai, 2020). Additionally, the meta-analysis results of Zych et al. (2017) show that empathy can significantly positively predict bystander defending. However, other studies found that empathy was not significantly related to bystander defending (Jenkins et al., 2016; Oh and Park, 2019), or that empathy was negatively correlated with bystander defending in school bullying (Barhight et al., 2013). Therefore, we proposed that empathy is positively correlated with bystander defending (Hypothesis 1).

### Moderators of Effect Sizes of Correlates of Defending

Furthermore, the inconsistencies regarding bystander defending and empathy may be because the type of empathy affects the relationship between empathy and bystander defending. Multi-dimensional researchers believe that empathy includes affective empathy and cognitive empathy. Affective empathy refers to the ability to experience the emotional state of others (Lovett and Sheffield, 2007). Cognitive empathy refers to recognizing other people's emotions and understanding their views (Hogan, 1969; Davis, 1983). Some studies showed that the relationship between both types of empathy and bystander defending is directionally inconsistent. For example, Barhight et al. (2013) found that affective empathy was negatively correlated with bystander defending, while Peets et al. (2015) found that cognitive empathy was positively correlated with bystander defending. Moreover, some studies have found that cognitive empathy could significantly predict bystander defending, while affective empathy had no significant effect (Espelage et al., 2012; Polanin et al., 2012). Contrarily, Wolfgang (2017) found that affective empathy was significantly correlated with bystander defending, while cognitive empathy had no significant effect. Finally, previous research indicates that affective empathy is more closely related to bystander defending than cognitive empathy (van der Ploeg et al., 2017; Fredrick et al., 2020). Therefore, we set forth Hypothesis 2: the magnitude of the association between empathy and bystander defending is moderated by the type of empathy.

Furthermore, the evaluators of defending behavior may also moderate the relationship between empathy and bystander defending. Bystander defending is measured using the Participant Role Questionnaire (PRQ) and its revised versions. The questionnaire was first compiled by Salmivalli et al. in 1996 and comprised 50 items. Salmivalli et al. revised the questionnaire in 1998 to 23 items applicable to middle school students (Salmivalli et al., 1998). Subsequently, in 2004, it was reduced to 15 items applicable to primary school students (Salmivalli and Voeten, 2004). Self-evaluation and evaluation by others may lead to inconsistent research results. Moreover, self-evaluation is affected by the social desirability effect, and subjects may thus report more defending behaviors. For example, Zhang (2005) found that the self-reported scores of junior high school students were significantly higher than peer-reported scores regarding defending; that is, they exaggerated their defending tendency in bullying situations. Several studies indicated that when self-evaluation was used to measure bystander defending (Nickerson and Mele-Taylor, 2014; Fredrick et al., 2020), defending behavior was higher than the correlation coefficient when using peer-evaluation (Gini et al., 2008; Wolfgang, 2017). Therefore, we suggested Hypothesis 3: the evaluation method of measuring defending behavior may moderate the association between empathy and bystander defending.

Different age groups will influence the correlation between empathy and bystander defending. For example, there is no significant correlation between empathy and bystander defending in early adolescence (Barhight et al., 2013); however, there is a significant correlation between empathy and bystander defending in middle adolescence (Correia and Dalbert, 2008). Additionally, Caravita et al. (2009) showed that the correlation coefficient between empathy and bystander defending increased proportionally with students' age. Moreover, the correlation coefficient between empathy and bystander defending among adolescents in middle school (Pozzoli et al., 2017; Yun and Graham, 2018) was stronger than for early adolescents (Pöyhönen et al., 2010; Lucas-Molina et al., 2018). Therefore, we proposed Hypothesis 4: the age of the subjects will moderate the relationship between empathy and bystander defending.

### Present Study

The main aim of this meta-analysis was to synthesize results from a large number of published and unpublished studies investigating relations between adolescent empathy and bystander defending in school bullying. We also examined potential moderators of these relations (e.g., the types of empathy, the evaluators of bystander defending, age of the sample). Building on the empathy-altruism hypothesis (Batson, 1987) and the theory of prosocial moral behavior and development (Hoffman, 2001), it is expected that empathy is positively correlated with bystander defending. Also, it is hypothesized that affective empathy is more closely related to bystander defending than cognitive empathy. Also, the relation between empathy and bystander defending assessed by self-report is stronger than bystander defending assessed by peer-report. Finally, the positive association between empathy and bystander defending can be assumed to be strengthened by age.

## Materials and Methods

### Search Strategies

We first conducted an electronic search of the following databases: Web of Science, ProQuest, CNKI, and WanFang Data using combinations of the relevant keywords: empathy^*^ AND defend^*^ or intervention^*^ or bully^*^. Since bystander defending behavior was proposed by Salmivalli in 1996, the retrieval time was set from 1996 to 2020. Moreover, we examined references cited in other articles using both backward and forward search methods. Based on the above-mentioned retrieval rules, 567 studies were ultimately retrieved.

**Figure 1:**
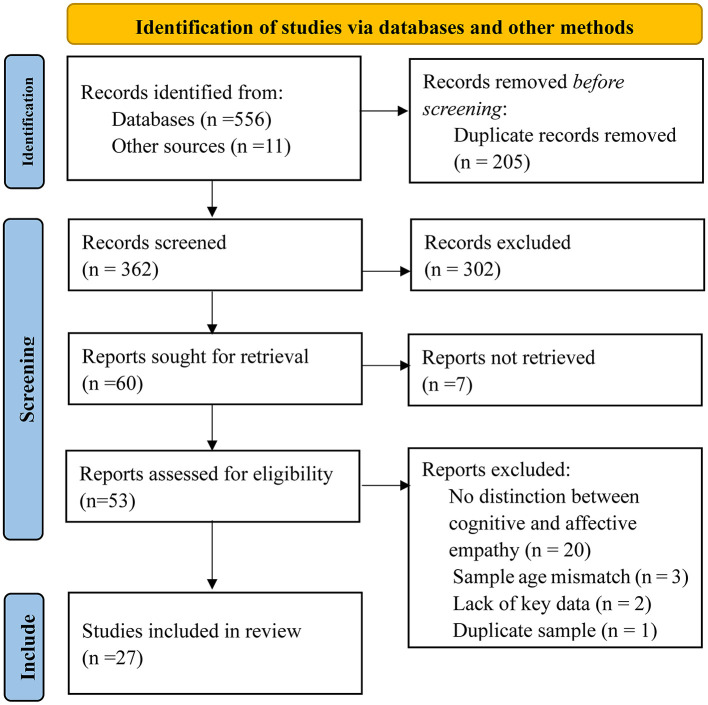
Flowchart presenting the selection process of studies.

Four criteria were used to screen the literature: (1) empirical studies on the relationship between empathy and traditional bullying bystander defending; (2) studies distinguishing between the cognitive and affective dimensions of empathy; (3) the necessary data were reported by meta-analysis, including sample size and effect size index, such as a correlation; and (4) the samples comprised school-age children and adolescents. First, 205 duplicate studies were eliminated by the title of the studies. Next, 302 irrelevant topics, e.g., cyberbullying studies, and non-empirical research studies were eliminated by reading the title and abstract. Next, seven articles cannot get the original text. Then, through full-text reading, a total of 26 pieces of literature were excluded, including 20 studies that did not distinguish between cognitive empathy and affective empathy, three studies that did not meet the sample requirements, two studies that did not have the necessary data for reporting, and one study with duplicate samples. Finally, 27 studies were included in the meta-analysis. Of these, six studies included multiple independent samples; thus, 35 independent studies were included in the meta-analysis. The detailed flowchart of the selection process for eligible studies is shown in Figure 1.

### Coding the Studies

The features of the research included in the meta-analysis were coded, including literature information (author name + publication year), sample size, adolescent development stage, average age, proportion of males, empathy type, evaluation subject of defending behavior, country, and correlation coefficient. When coding adolescent development stages, we found that some studies reported only the grades of the subjects rather than the average age. Thus, the adolescent development stages were divided into early, middle and late stages according to the age and grades of the subjects. The early stage refers to subjects aged under 12 years or in primary school, the middle stage refers to subjects aged 12~14 years or in junior high school, and the late stage refers to subjects aged over 15 years old or in high school. Since only one included study comprised subjects belonging to the late adolescent stage, the research subjects were coded as the middle stage.

In this study, the first author created the research feature coding table, read the original literature, and completed the coding table prepared in advance. After verification and proofreading by other authors, the results indicated no obvious difference between the two codes, except for a few data deviations. See Table 1 for the coding data included in the original study.

**Table 1:** Studies included in the meta-analysis.

**References**	**Country**	***r***	***N***	**Male ratio%**	**average age**	**Empathy type**	**Developmental stage**	**Defending behavior evaluators**
Barchia and Bussey (2011)	Australia	0.24	1167	47.47	12	Affective	Middle	Self
Barhight et al. (2013)	United States	−0.17	771	46.17	10.6	Affective	Early	Peer
Caravita et al. (2009)	Italy	0.16	130	100	9.3	Affective	Early	Peer
	Italy	0.06	130	100	9.3	Cognitive	Early	Peer
	Italy	0.23	136	0	9.3	Affective	Early	Peer
	Italy	0.00	136	0	9.3	Cognitive	Early	Peer
	Italy	0.37	104	100	12.4	Affective	Middle	Peer
	Italy	0.14	104	100	12.4	Cognitive	Middle	Peer
	Italy	0.05	91	0	12.4	Affective	Middle	Peer
	Italy	0.14	91	0	12.4	Cognitive	Middle	Peer
Caravita et al. (2010)	Italy	0.19	98	100	10.2	Affective	Early	Peer
	Italy	0.25	113	0	10.2	Affective	Early	Peer
Carroll (2014)	United States	0.27	282	30.85	12.8	Affective	Middle	Self
Correia and Dalbert (2008)	Portugal	0.50	187	51.87	14.5	Affective	Middle	Self
Cuervo et al. (2018)	Mexico	0.36	1224	45.9	13.5	Affective	Middle	Self
Dollar (2016)	United States	0.20	207	43	12.7	Affective	Middle	Self
	United States	0.23	207	43	12.7	Cognitive	Middle	Self
Espelage et al. (2012)	United States	0.41	168	100	NA	Affective	Middle	Self
	United States	0.52	168	100	NA	Cognitive	Middle	Self
	United States	0.40	179	0	NA	Affective	Middle	Self
	United States	0.33	179	0	NA	Cognitive	Middle	Self
Fredrick et al. (2020)	United States	0.28	336	58.93	NA	Affective	Early	Self
	United States	0.32	336	58.93	NA	Cognitive	Early	Self
Gini et al. (2007)	Italy	0.22	176	100	13.2	Affective	Middle	Peer
	Italy	0.10	176	100	13.2	Cognitive	Middle	Peer
	Italy	0.17	142	0	13.2	Affective	Middle	Peer
	Italy	0.16	142	0	13.2	Cognitive	Middle	Peer
Gini et al. (2008)	Italy	0.14	294	52.8	13.3	Cognitive	Middle	Peer
	Italy	0.17	294	52.8	13.3	Affective	Middle	Peer
Lucas-Molina et al. (2018)	Spain	0.10	2050	49.2	9.8	Affective	Early	Peer
Ma (2020)	Taiwan, China	0.21	730	51	12.8	Affective	Middle	Self
Menolascino and Jenkins (2018)	United States	0.12	179	0	NA	Affective	Middle	Self
	United States	0.16	179	0	NA	Cognitive	Middle	Self
	United States	0.36	167	100	NA	Affective	Middle	Self
	United States	0.17	167	100	NA	Cognitive	Middle	Self
Nickerson and Mele-Taylor (2014)	United States	0.37	262	46.18	12.2	Affective	Middle	Self
Oh and Park (2019)	Korea	0.07	163	47.23	NA	Affective	Middle	Self
Peets et al. (2015)	Finland	0.30	6708	49.00	NA	Affective	Early	Peer
	Finland	0.17	6708	49.00	NA	Cognitive	Early	Peer
Pöyhönen et al. (2010)	Finland	0.12	489	47.44	12.3	Affective	Early	Peer
	Finland	0.10	489	47.44	12.3	Cognitive	Early	Peer
Pozzoli et al. (2017)	Italy	0.54	398	52.76	12.3	Affective	Middle	Self
	Italy	0.49	398	52.76	12.3	Cognitive	Middle	Self
Rieffe and Camodeca (2016)	Italy	0.26	182	46.7	13.4	Affective	Middle	Peer
	Italy	0.22	182	46.7	13.4	Cognitive	Middle	Peer
van Beurden et al. (2012)	Netherlands	0.39	92	30.43	15.8	Affective	Middle	Self
van der Ploeg et al. (2017)	Finland	0.31	4209	50	11.3	Affective	Early	Peer
	Finland	0.18	4209	50	11.3	Cognitive	Early	Peer
Wolfgang (2017)	United States	0.17	322	34.78	13.4	Affective	Middle	Peer
	United States	0.05	322	34.78	13.4	Cognitive	Middle	Peer
Yun and Graham (2018)	Korea	0.24	828	100	14.0	Affective	Middle	Peer
	Korea	0.24	828	100	14.0	Cognitive	Middle	Peer
	Korea	0.24	545	0	14.0	Affective	Middle	Peer
	Korea	0.16	545	0	14.0	Cognitive	Middle	Peer
Li et al. (2020)	China	0.15	912	56.58	NA	Affective	Middle	Self
	China	0.22	912	56.58	NA	Cognitive	Middle	Self
Ma (2018)	China	0.59	971	NA	NA	Affective	Early	Self
	China	0.54	971	NA	NA	Cognitive	Early	Self

*N, number of participants; NA, the unreported average age*.

### Analyses

Pearson's product-moment correlation coefficient was used to determine the effect size. To eliminate the influence of different sample sizes, the correlation coefficients of each study were transformed by Fisher's Z, and the average number of Z values after transformation were calculated. The average number of Z values were then transformed into a correlation coefficient (Lipsey and Wilson, 2001).

The outliers were identified by studentized deleted residuals. Values >2.5 were identified as outliers (Deng et al., 2016). We used Cook's distance and standardized *Df Beta* to identify the threatening effect size, which were values >1 (Deng et al., 2016). Research results which had abnormal values and posed a threat to the effect quantity were deleted, and other effect sizes were retained for subsequent analyses.

A three-level random effect model was used to estimate the total effect. Some studies use the same sample to report multiple effects, and the effects from the same study tend to be more similar than those from different studies, which violates the assumption of the independence of effect sizes (Cheung, 2014). In the past, meta-analyses addressed these limitations by deleting parts of effect sizes or combining effect sizes; however, these cause information loss or discount the differences between effect sizes, respectively. Therefore, we adopted the three-level random effect model, which decomposes the total variance of effect size into sampling variance (level 1), the variance between effect sizes from the same study (level 2), and the variance between studies (level 3) (Van den Noortgate et al., 2013). The three-level random effect model can include the effects from the same research and all available effects to obtain the maximum information and statistical ability.

The funnel plot and Egger's regression method were used to test the publication bias. Some studies have shown that published research is likelier to present significant results than unpublished research, and the meta-analysis includes five dissertations. Thus, it is necessary to evaluate the publishing deviation (Rothstein et al., 2005). Egger linear regression analysis is used to test the symmetry of the funnel plot and significant results indicate publication bias, wherein the trim and fill method is used to analyze sensitivity and correct total effect sizes.

The likelihood ratio test (*LRT*) and variance distribution of effect sizes were used to test whether heterogeneity exists (Van den Noortgate et al., 2013). If the result of *LRT* is significant or the sampling variance accounts for <75% of the total variance, then heterogeneity is confirmed, and it is reasonable to conduct moderator analysis.

The Omnibus Test under the fixed-effect model was used for moderator analysis (Van den Noortgate et al., 2013). For two levels of classified moderator variables, the moderator variables are converted into two virtual variables, and each virtual variable is set at 0/1. The omnibus test follows F-distribution and a significant result indicates that the moderator variables are significant. T-distribution was used to test the effect sizes of each level and whether the difference between them was significant (Raudenbush and Bryk, 2002).

The analyses were conducted using the metafor package for the R environment (Viechtbauer, 2010).

## Results

### Analysis of Outliers

The results showed that studentized deleted residuals of the second and the 57th effects were 2.88 and 2.96, respectively, and were classified as outliers. The standardized *Df Beta* values of the second and the 57th effect quantity were 1.92 and 1.94, respectively, both of which were threatening effect values. Thus, these two effect sizes were deleted, and other effect sizes were retained for meta-analysis.

### Analysis of Heterogeneity

*LRT* showed that when comparing the entire model with the model after deleting level 2, there was a significant difference between the effects in the study (σ^2^ = 0.003, *LRT* = 90.44, *p* < 0.0001). When comparing the whole model with the model after deleting level 3, there were significant differences between the studies (σ^2^ = 0.015, *LRT* = 12.70, *p* < 0.001). Therefore, the whole model including levels 2 and 3 were selected for this study. Additionally, the variance distribution results showed that the variance of level 2 accounted for 16.46% and the variance of level 3 accounted for 76.13%. Thus, the total systematic error accounted for 92.59%. Considering these results, it is necessary to investigate the influence of moderator variables on the relationship between them.

### Publication Bias and Main Effect Sizes Analysis

The funnel plot showed that the effect sizes were concentrated above the graph and evenly distributed on both sides of the total effect. The Egger's linear regression results were not significant (*t* = 0.18, *df* = 54, *p* = 0.86). This indicates that there is no significant publication bias in this study, and the results of the meta-analysis are reliable.

The random-effect model was used to estimate the correlation coefficient between empathy and bystander defending behavior. The results showed that the correlation between empathy and defending was 0.25 (*CI* = 0.21 ~ 0.30, *p* < 0.001). Gignac and Szodorai (2016) proposed that 0.1 < *r* < 0.2 signifies a low correlation, 0.2 ≤ *r* ≤ 0.3 indicates a medium correlation, and 0.3 < *r* shows a strong correlation. Thus, there was a moderate correlation between empathy and bystander defending behavior.

### Analysis of Moderator

The omnibus test was used to examine the moderating effects of empathy type, developmental stage, and defending behavior evaluators on the relationship between empathy and bystander defending. The results showed that empathy type significantly moderated the relationship between empathy and bystander defending, [*F*_(1,54)_ = 6.31, *p* = 0.02]. The correlation coefficient between affective empathy and bystander defending was significantly stronger than for cognitive empathy and defending. The evaluator of bystander defending behavior significantly moderated the relationship between empathy and defending, [*F*_(1, 54)_ = 15.18, *p* < 0.001]. Self-evaluation of defending behavior showed a greater correlation coefficient between empathy and bystander defending than for peer evaluation. The subjects' developmental stage did not act as significant moderators, [*F*_(1,54)_ = 0.45, *p* = 0.05]. Specific results are shown in Table 2.

**Table 2:** Results for the moderators of empathy and defending.

**Moderators**	***k***	**Fisher's Z (95%CI)**	**Difference (95%CI)**	***t***	***r***	**Omnibus test**	**Level-2 variance**	**Level-3 variance**
Empathy type						6.31^*^	0.002	0.017
Affective	23	0.27 (0.22, 0.32)		10.67^***^	0.27			
Cognitive	23	0.22 (0.17, 0.28)	−0.05 (0.10, 0.01)^*^	7.94^***^	0.22			
Defending behavior evaluators						15.18^***^	0.003	0.009
Peer	31	0.18 (0.12, 0.24)		6.31^***^	0.18			
Self	25	0.34 (0.28, 0.40)	0.16 (0.08, 0.24)^***^	11.62^***^	0.32			
Developmental stage						0.45	0.003	0.015
Early	16	0.23 (0.14, 0.32)		9.15^***^	0.26			
Middle	40	0.27 (0.21, 0.33)	0.04 (−0.07, 0.14)	5.13^***^	0.23			

*k, number of effect sizes*.

* *p < 0.05*,

*** *p < 0.001*.

## Discussion

### Empathy and Defending

A total of 35 independent sample studies were included in the current study to perform a meta-analysis regarding the correlation between empathy and bystander defending behavior. Our results supported Hypothesis 1; that is, there was a significant positive correlation between empathy and bystander defending behavior. However, the hypotheses of zero correlation and negative correlation were not supported. The results are consistent with previous research (Van Noorden et al., 2015; Zych et al., 2017; Ma et al., 2019), as well as the meta-analysis results of Nickerson et al. (2015). However, the correlation coefficient obtained in this meta-analysis was lower than that obtained by Nickerson et al. (2015), which may be related to the inclusion criteria for the literature selection. First, compared with Nickerson et al. (2015), we included additional research from 2015 to 2020, and the number of studies may have influenced the results. Secondly, although the meta-analysis of Nickerson et al. (2015) distinguished between cognitive and affective empathy in the literature inclusion criteria, some studies which were included did not actually classify empathy, which may also lead to inconsistent results.

Ettekal et al. (2015) emphasized the importance of considering affective processes (such as affective understanding and empathy) when studying bullying and bystander behavior, and posited that affective processes affect children's social cognition and thus their social goals. Empathy is conceptualized as an affective feature and a cognitive ability (Davis, 1983; Jolliffe and Farrington, 2006). However, empathy is a necessary but insufficient component in the development of prosocial behavior (Jolliffe and Farrington, 2006). Specifically, empathy elicits the emotional experience of the victim within the individual. Therefore, adolescents with a higher level of empathy are likelier to recognize the victim's feelings and needs, and thus enact defending behavior. Several previous studies have also proven that defending was related to higher empathy. Therefore, enhancing adolescents' empathy is essential in improving their bystander defending behavior in school bullying situations.

### Moderation Effects

We found that empathy type moderated the relationship between empathy and bystander defending. Although both cognitive empathy and affective empathy were significantly associated with bystander defending, the correlation between affective empathy and bystander defending was stronger, supporting Hypothesis 2. Past researchers assumed that the cognitive and affective components of empathy were different; thus, cognitive and affective empathy should be considered together (Davis, 1983; Jolliffe and Farrington, 2004). This is consistent with our findings. For example, a review by Van Noorden et al. (2015) also found that affective empathy had a higher correlation with bystander defending than did cognitive empathy. However, our results are inconsistent with the meta-analysis results of Nickerson et al. (2015). This may be because the outliers were not deleted in the meta-analysis of Nickerson et al. (2015), which may reduce the correlation coefficient between the overall affective empathy and bystander defending, leading to empathy types that did not yield moderator effects. Fredrick et al. (2020) examined the relationship between cognitive and affective empathy and five proposed stages of defending in bullying. They found that cognitive empathy was significantly positively correlated with three stages: paying attention to a school bullying incident, undertaking intervention responsibility, and knowing how to deal with a bullying incident. However, affective empathy was significantly positively correlated with interpreting the situation as an emergency and the actual defending behavior. This indicated that while cognitive empathy is important, affective empathy is necessary to spur participation in defending behavior. Compared with affective empathy, cognitive empathy had a weaker correlation with bystander defending; however, it had a positive correlation with bullying behavior. According to previous studies, general empathy includes affective and cognitive components and does not distinguish between defender and outsider (Gini et al., 2008). However, individuals who manipulate situations and other people for their benefit must undergo advanced psychological skills training, including perspective-taking skills and social intelligence to understand and predict others' behaviors (Caravita et al., 2009). Thus, feeling another person's emotions (affective empathy) is likelier to promote positive behavior (defending), while understanding another person's affect (cognitive empathy) may be used to harm that person (Pöyhönen et al., 2010).

In the current study, we found that the evaluators of defending behavior significantly regulated the relationship between empathy and bystander defending, supporting Hypothesis 3. While the correlation between empathy and bystander defending was significant for both self-evaluation and peer-evaluation, it was significantly stronger for self-evaluation. This may be because when self-evaluation is adopted, individuals may readily report defending behaviors. When individuals evaluate their behavior, they will consciously conceal behaviors that do not conform to social expectations, while exaggerating socially desirable behaviors to maintain their image and self-esteem. Ma et al. (2019) suggested that using the self-report method with a clear description of the imaginary victims can better stimulate teenagers' self-identity to the victims, thus enhancing their willingness to engage in defending. Compared research reporting on actual defending behavior, this tendency may exaggerate the effect size between affective empathy and bystander defending. According to previous studies, compared with self-reported evaluations, the comprehensive judgment of peer students may be a more accurate measurement (Bouman et al., 2012; Hunt et al., 2012). However, there are some constraints regarding peer evaluation because peers may not accurately identify the behavior of individuals in school bullying, and the evaluation of peers is also affected by social pressure. Therefore, future research must employ various methods of reporting to address these inherent limitations.

We found no significant moderating effect of the developmental stage on the relationship between empathy and bystander defending; thus, Hypothesis 4 was unsupported. The correlation between empathy and bystander defending in early adolescence was not significantly different from that of middle adolescence; however, the correlation between empathy and bystander defending in early and middle adolescence was significant. Previous studies suggested that young children were likelier to engage in more defending behavior than older children (Salmivalli and Voeten, 2004; Evans and Smokowski, 2015). Furthermore, Ma et al. (2019) found that younger children were likelier to report defending behavior or to be nominated as a defender than older children. Simultaneously, some studies propose that empathy decreases with age (Phillips et al., 2002). Thus, this may explain why there was no significant difference in the correlation coefficient between empathy and bystander defending behavior in early and middle adolescence. Furthermore, because some studies included in the meta-analysis only reported the grade of the subjects, we divided the adolescent age into early and middle stages according to the grade and age. This general classification method may lead to excessive loss of age information, and if the age of the subjects is considered as a continuous variable for moderator analysis, different results may be obtained.

### Limitations

Since most studies included in the meta-analysis did not report the correlation between empathy and the defending behavior of male and female subjects respectively, we did not analyze the moderating effect of gender. Compared with boys, girls tend to participate more in defending behavior (O'Connell et al., 1999; Pozzoli and Gini, 2010; Espelage et al., 2012; van der Ploeg et al., 2017). Several studies have found that girls have a higher level of empathy than boys (Eisenberg and Lennon, 1983; Pöyhönen et al., 2010; Van der Graaff et al., 2014). Therefore, future research can further examine the regulatory role of gender. Additionally, this study could not include all unpublished studies and non-English and Chinese studies and excluded some studies that did not provide effect sizes. This may have led to the loss of some samples. Therefore, the unpublished gray literature can be further included in future research to expand the number of studies.

### Conclusion

Adolescent empathy can significantly positively predict bystander defending behavior in school bullying. The relationship is moderated by empathy type and the evaluator of the defending behavior. Furthermore, the correlation between affective empathy and defending is significantly stronger than that between cognitive empathy and defending, and the correlation between empathy and self-evaluative defending was significantly stronger than that between empathy and peer evaluated defending. Nevertheless, empathy type and the evaluator of the defending behavior have to be taken into account and controlled for in future studies. Thus, teaching students how to empathize with others may be crucial for adolescents to engage in defending behavior toward victims. Teaching students to be more aware of emotional distress of others and taking the “emotional” perspective of the victim may enhance affective empathy to aid youth interpreting bullying as a distressing event that requires intervention. This may increase cognitive empathy by teaching them how to recognize certain emotions (e.g., humiliation, fear) and what types of scenarios may elicit such emotions (e.g., a friend joking with another friend would likely not make one individual feel embarrassed).

## Data Availability Statement

The original contributions presented in the study are included in the article/supplementary material, further inquiries can be directed to the corresponding author.

## Author Contributions

XD conceived and designed the study. JY and YW collected data. XD and JY analyzed the data and wrote the paper. All authors contributed to the article and approved the submitted version.

## Conflict of Interest

The authors declare that the research was conducted in the absence of any commercial or financial relationships that could be construed as a potential conflict of interest.

## Publisher's Note

All claims expressed in this article are solely those of the authors and do not necessarily represent those of their affiliated organizations, or those of the publisher, the editors and the reviewers. Any product that may be evaluated in this article, or claim that may be made by its manufacturer, is not guaranteed or endorsed by the publisher.
